# How Many Scientists Fabricate and Falsify Research? A Systematic Review and Meta-Analysis of Survey Data

**DOI:** 10.1371/journal.pone.0005738

**Published:** 2009-05-29

**Authors:** Daniele Fanelli

**Affiliations:** INNOGEN and ISSTI-Institute for the Study of Science, Technology & Innovation, The University of Edinburgh, Edinburgh, United Kingdom; University of Exeter, United Kingdom

## Abstract

The frequency with which scientists fabricate and falsify data, or commit other forms of scientific misconduct is a matter of controversy. Many surveys have asked scientists directly whether they have committed or know of a colleague who committed research misconduct, but their results appeared difficult to compare and synthesize. This is the first meta-analysis of these surveys.

To standardize outcomes, the number of respondents who recalled at least one incident of misconduct was calculated for each question, and the analysis was limited to behaviours that distort scientific knowledge: fabrication, falsification, “cooking” of data, etc… Survey questions on plagiarism and other forms of professional misconduct were excluded. The final sample consisted of 21 surveys that were included in the systematic review, and 18 in the meta-analysis.

A pooled weighted average of 1.97% (N = 7, 95%CI: 0.86–4.45) of scientists admitted to have fabricated, falsified or modified data or results at least once –a serious form of misconduct by any standard– and up to 33.7% admitted other questionable research practices. In surveys asking about the behaviour of colleagues, admission rates were 14.12% (N = 12, 95% CI: 9.91–19.72) for falsification, and up to 72% for other questionable research practices. Meta-regression showed that self reports surveys, surveys using the words “falsification” or “fabrication”, and mailed surveys yielded lower percentages of misconduct. When these factors were controlled for, misconduct was reported more frequently by medical/pharmacological researchers than others.

Considering that these surveys ask sensitive questions and have other limitations, it appears likely that this is a conservative estimate of the true prevalence of scientific misconduct.

## Introduction

The image of scientists as objective seekers of truth is periodically jeopardized by the discovery of a major scientific fraud. Recent scandals like Hwang Woo-Suk's fake stem-cell lines [1] or Jan Hendrik Schön's duplicated graphs [2] showed how easy it can be for a scientist to publish fabricated data in the most prestigious journals, and how this can cause a waste of financial and human resources and might pose a risk to human health. How frequent are scientific frauds? The question is obviously crucial, yet the answer is a matter of great debate [3], [4].

A popular view propagated by the media [5] and by many scientists (e.g. [6]) sees fraudsters as just a “few bad apples” [7]. This pristine image of science is based on the theory that the scientific community is guided by norms including disinterestedness and organized scepticism, which are incompatible with misconduct [8], [9]. Increasing evidence, however, suggests that known frauds are just the “tip of the iceberg”, and that many cases are never discovered. The debate, therefore, has moved on to defining the forms, causes and frequency of scientific misconduct [4].

What constitutes scientific misconduct? Different definitions are adopted by different institutions, but they all agree that fabrication (invention of data or cases), falsification (wilful distortion of data or results) and plagiarism (copying of ideas, data, or words without attribution) are serious forms of scientific misconduct [7], [10]. Plagiarism is qualitatively different from the other two because it does not distort scientific knowledge, although it has important consequences for the careers of the people involved, and thus for the whole scientific enterprise [11].

There can be little doubt about the fraudulent nature of fabrication, but falsification is a more problematic category. Scientific results can be distorted in several ways, which can often be very subtle and/or elude researchers' conscious control. Data, for example, can be “cooked” (a process which mathematician Charles Babbage in 1830 defined as “an art of various forms, the object of which is to give to ordinary observations the appearance and character of those of the highest degree of accuracy”[12]); it can be “mined” to find a statistically significant relationship that is then presented as the original target of the study; it can be selectively published only when it supports one's expectations; it can conceal conflicts of interest, etc… [10], [11], [13], [14], [15]. Depending on factors specific to each case, these misbehaviours lie somewhere on a continuum between scientific fraud, bias, and simple carelessness, so their direct inclusion in the “falsification” category is debatable, although their negative impact on research can be dramatic [11], [14], [16]. Henceforth, these misbehaviours will be indicated as “questionable research practices” (QRP, but for a technical definition of the term see [11]).

Ultimately, it is impossible to draw clear boundaries for scientific misconduct, just as it is impossible to give a universal definition of professional malpractice [10]. However, the intention to deceive is a key element. Unwilling errors or honest differences in designing or interpreting a research are currently not considered scientific misconduct [10].

To measure the frequency of misconduct, different approaches have been employed, and they have produced a corresponding variety of estimates. Based on the number of government confirmed cases in the US, fraud is documented in about 1 every 100.000 scientists [11], or 1 every 10.000 according to a different counting [3]. Paper retractions from the PubMed library due to misconduct, on the other hand, have a frequency of 0.02%, which led to speculation that between 0.02 and 0.2% of papers in the literature are fraudulent [17]. Eight out of 800 papers submitted to *The Journal of Cell Biology* had digital images that had been improperly manipulated, suggesting a 1% frequency [11]. Finally, routine data audits conducted by the US Food and Drug Administration between 1977 and 1990 found deficiencies and flaws in 10–20% of studies, and led to 2% of clinical investigators being judged guilty of serious scientific misconduct [18].

All the above estimates are calculated on the number of frauds that have been discovered and have reached the public domain. This significantly underestimates the real frequency of misconduct, because data fabrication and falsification are rarely reported by whistleblowers (see Results), and are very hard to detect in the data [10]. Even when detected, misconduct is hard to prove, because the accused scientists could claim to have committed an innocent mistake. Distinguishing intentional bias from error is obviously difficult, particularly when the falsification has been subtle, or the original data destroyed. In many cases, therefore, only researchers know if they or their colleagues have wilfully distorted their data.

Over the years, a number of surveys have asked scientists directly about their behaviour. However, these studies have used different methods and asked different questions, so their results have been deemed inconclusive and/or difficult to compare (e.g. [19], [20]). A non-systematic review based on survey and non-survey data led to estimate that the frequency of “serious misconduct”, including plagiarism, is near 1% [11].

This study provides the first systematic review and meta-analysis of survey data on scientific misconduct. Direct comparison between studies was made possible by calculating, for each survey question, the percentage of respondents that admitted or observed misconduct at least once, and by limiting the analysis to qualitatively similar forms of misconduct -specifically on fabrication, falsification and any behaviour that can distort scientific data. Meta-analysis yielded mean pooled estimates that are higher than most previous estimates. Meta-regression analysis identified key methodological variables that might affect the accuracy of results, and suggests that misconduct is reported more frequently in medical research.

## Methods

### Searching

Electronic resources were searched during the first two weeks of August 2008. Publication and journal databases were searched in English, while the Internet and resources for unpublished and “grey” literature were searched using English, Italian, French and Spanish words.

#### Citation databases

The Boolean string “research misconduct” OR “research integrity” OR “research malpractice” OR “scientific fraud” OR “fabrication, falsification” OR “falsification, fabrication” was used to search: Science Citation Index Expanded (SCI-EXPANDED), Social Sciences Citation Index (SSCI), Arts & Humanities Citation Index (A&HCI), Conference Proceedings Citation Index- Science (CPCI-S), BIOSIS Previews, MEDLINE, Business Source Premier, CINAHL Plus, SPORTDiscus, Library, Information Science & Technology Abstracts, International Bibliography of the Social Sciences, America: History & Life, Teacher Reference Center, Applied Social Sciences Index And Abstracts (ASSIA), ERIC, Index Islamicus, CSA linguistics and language behaviour, Physical Education Index, PILOTS, Social Services Abstracts, Sociological Abstracts, Proquest Dissertation & Theses, ECONLIT, Educational Research Abstracts (ERA) Online, Article First, Economic and Social Data Service, Francis, Geobase, Georefs, Global Health (CABI), Index to Theses, International Bibliography of the Social Sciences (IBSS), IEEE Xplore, INSPEC, JSTOR, Mathematical Sciences Net (MathSciNet), PubMEd, Russian Academy of Sciences bibliographies, Sciencedirect, Teacher Reference Center, EMBASE, EMBASE Classics, PSYCHINFO.

#### Scientific journals

The Boolean string “research misconduct” OR “research integrity” OR “research malpractice” OR “scientific fraud” OR “fabrication, falsification” OR “falsification, fabrication” was used to search: Interdisciplinary Science Reviews, American Journal of Sociology, Annual Review of Sociology, PNAS, Issues in Science & Technology, Journal of Medical Ethics, PLoSONE, Science and Engineering Ethics, Sociology of Health & Illness, Minerva, The Scientific World Journal, Social Science Research, Social Studies of Science, Science in Context.

#### Grey literature databases

The Boolean string “research misconduct” OR “research integrity” OR “research malpractice” OR “scientific fraud” OR “fabrication, falsification” OR “falsification, fabrication” was used to search: SIGLE, National Technical Information Service, British Library Collections, British Library Direct, Canadian Evaluation Society, Bioethics Literature Database.

The Italian string “etica AND ricerca” was used in: CNR database.

The French string “scientifique AND “ethique” OR “fraude” OR “faute” OR “enquete” OR “sondage” was used in: LARA -Libre acces aux rapports scientifiques et techiques

#### Internet search engines

The Boolean string “research misconduct” OR “research integrity” OR “research malpractice” OR “scientific fraud” OR “fabrication, falsification” OR “falsification, fabrication”, the Spanish Boolean string “ética cientifica” OR “faltas éticas” the French Boolean string “faute scientifique” OR “éthique scientifique” were used to search: ScienceResearch.com, Scirus.

Titles and available abstracts of all records were examined, and the full text of all potentially relevant studies was retrieved. The references list of the retrieved studies and of other documents was also examined in search of potentially relevant papers.

### Selection

Only quantitative survey data assessing how many researchers have committed or observed colleagues committing scientific misconduct in the past were included in this review. Surveys asking only opinions or perceptions about the frequency of misconduct were not included.

To allow direct quantitative comparison across data sets, studies were included only if they presented data in frequency or percentage categories, one of which was a “never” or “none” or “nobody” category - indicating that the respondent had never committed or observed the behaviour in question. Studies lacking such a category, or presenting results in statistical formats that prevented the retrieval of this information (e.g. mean and standard deviation) were excluded. Respondents of any professional position and scientific discipline were included, as long as they were actively conducting publishable research, or directly involved in it (e.g. research administrators). Surveys addressing misconduct in undergraduate students were excluded, because it was unclear if the misconduct affected publishable scientific data or only scholastic results.

This review focused on all and only behaviours that can falsify or bias scientific knowledge through the unjustified alteration of data, results or their interpretation (e.g. any form of fabrication and falsification, intentional non-publication of results, biased methodology, misleading reporting, etc…). Plagiarism and professional misconduct (e.g. withholding information from colleagues, guest authorship, exploitation of subordinates etc…) were excluded from this review. Surveys that made no clear distinction between the former and latter types of misconduct (e.g. that asked about fabrication, falsification and plagiarism in the same question) were excluded.

Any available data on scientists' reaction to alleged cases of misconduct was extracted from included studies. Since these data provided only additional information that was not the focus of the review, survey questions that did not distinguish between data manipulation and plagiarism were included in this section of the results, but clearly identified.

### Validity assessment

Surveys that did not sample respondents at random, or that did not provide sufficient information on the sampling methods employed where given a quality score of zero and excluded from the meta-analysis. All remaining papers were included, and were not graded on a quality scale, because the validity and use of quality measures in meta-analysis is controversial [21], [22]. Instead of using an arbitrary measure of quality, the actual effect of methodological characteristics on results was tested and then controlled for with regression analysis. In the tables listing study characteristics, the actual words reported in the paper by the authors are quoted directly whenever possible. The few cases where a direct quotation could not be retrieved are clearly indicated.

### Data abstraction

For each question, the percentage of respondents who recalled committing or who observed (i.e. had direct knowledge of) a colleague who committed one or more times the specified behaviour was calculated. In the majority of cases, this required summing up the responses in all categories except the “none” or “never” category, and the “don't know” category.

Some studies subdivided the sample of respondents according to a variety of demographic characteristics (e.g. gender, career level, professional position, academic discipline, etc…) and disaggregated the response data accordingly. In all these cases, the data was re-aggregated.

Given the objectivity of the information collected and the fact that all details affecting the quality of studies are reported in this paper, it was not necessary to have the data extracted/verified by more than one person.

### Quantitative data synthesis

The main outcome of the meta-analysis was the percentage (proportion) of respondents that recalled committing or that knew of a colleague committing the specified behaviour at least once in the given recall period. This measure was not normally distributed (Kolmogorov-Smirnov test: 0.240, df = 19, P = 0.005) so it was *logit* transformed [23], and weighted by inverse variance of logit transformed proportion using the following equations for effect size, standard error and weight, respectively:
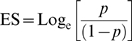


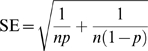


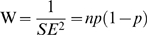
Where *p* is the proportion of respondents recalling at least one case of the specified behaviour, and n is the total number of respondents. The distribution of the logit-transformed effect sizes was not significantly different from normal (K-S: 0.109, df = 19, P = 0.2). To facilitate their interpretation, the final logit results (ES and 95%CI) were back-transformed in percentages using the following equations for proportion and percentages, respectively:
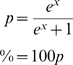
Where *x* is either ES or each of the corresponding 95%CI values.

Mean pooled effect size was calculated assuming a random effects model, and homogeneity was tested with Chochran's Q test. Differences between groups of studies were tested using inverse variance weighted one-way ANOVA. The combined effect of independent variables on effect sizes was tested with inverse variance weighted regression assuming a random effects model and estimated via iterative maximum likelihood.

To avoid the biasing effect of multiple outcomes within the same study, all meta-analyses on the main outcome of interest (i.e. the prevalence of data fabrication, falsification and alteration) were conducted using only one outcome per study. For the same reason, in the regression analysis, which combined all available effect sizes on data fabrication, falsification and alteration, studies that had data both on self- and on non self- where used only for the former.

The regression model first tested the combined effect of three methodological factors measured by binary variables (self- vs non-self- reports, handed vs mailed questionnaire, questions using the word “falsification” or “fabrication” vs questions using “alteration”, “modification” etc…). Then, the effect of several study characteristics was tested (year when the survey was conducted, surveys conducted in the USA vs anywhere else, surveys conducted exclusively on researchers vs any other, biomedical vs other types of research, social sciences vs natural sciences, medical consultants and practitioners vs other). To avoid over-fitting, each study characteristic was tested independently of the others.

Questions on behaviours of secondary interest (questionable research practices) where too diverse to allow meaningful meta-analysis, so they were combined in broad categories for which only crude unweighted parameters were calculated. All statistical analyses were run on SPSS software package. Meta-analyses were conducted using the “MeanES”, “MetaF” and “MetaReg” macros by David B. Wilson [24].

### Publication bias-Sensitivity analysis

The popular funnel-plot-based methods to test for publication bias in meta-analysis are inappropriate and potentially misleading when the number of included studies is small and heterogeneity is large [25], [26]. However, the robustness of results was assessed with a sensitivity analysis. Pooled weighted estimates for effect size and regression parameters were calculated leaving out one study at a time, and then compared to identify influential studies. In addition, to further assess the robustness of conclusions, meta-analyses and meta-regression were run without logit transformation.

## Results

### Flow of included studies

Electronic search produced an initial list of 3276 references. Examination of titles and abstracts, and further examination of the references lists in the retrieved papers and in other sources led to a preliminary list of 69 potentially relevant studies. Of these, 61 were published in peer-reviewed journals, three were dissertations theses, three were published in non-peer reviewed popular science magazines, one was published in a book chapter, and one was published in a report. All studies were published in English except for one in Spanish.

**Figure 1:**
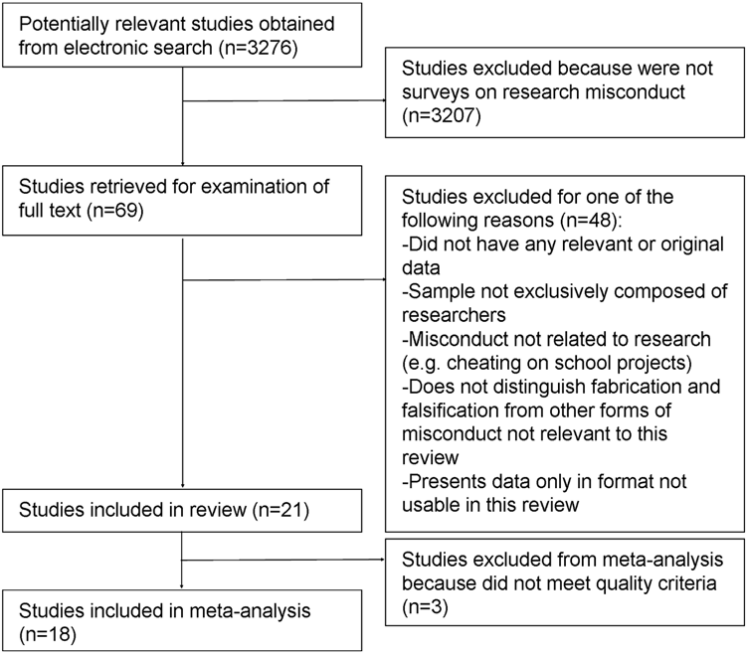
Study selection flow diagram.

After examination of full text, 33 studies were excluded because they did not have any relevant or original data, two because they presented data exclusively in a format that could not be used in this review (e.g. means and standard deviations), eight because their sample included non-researchers (e.g. students) and/or because they addressed forms of academic misconduct not directly related to research (e.g. cheating on school projects), five because they do not distinguish fabrication and falsification from types of misconduct not relevant to the scopes of this review (Table S1). Therefore, 21 studies were included in the review. Three of these did not match the quality requirements to be included in the meta-analysis. Data from these three studies was only used to estimate crude unweighted means for QRP and more generic questions, and not for analyzing the main outcome of interest (data fabrication, falsification, modification). Therefore, the meta-analysis was conducted on 18 studies (Figure 1).

### Study characteristics

Table 1 lists the characteristics of included studies and their quality score for inclusion in meta-analysis. Included surveys were published between 1987 and 2008, but had been conducted between 1986 ca and 2005. Respondents were based in the United States in 15 studies (71% ca of total), in the United Kingdom in 3 studies (14% ca), two studies had a multi-national sample (10% ca) and one study was based in Australia. Six studies had been conducted among biomedical researchers, eight were more specifically targeted at researchers holding various positions in the medical/clinical sciences (including pharmacology, nursing, health education, clinical biostatistics, and addiction-studies), six surveys had multi-disciplinary samples, one surveyed economists.

**Table 1:** Characteristics of studies included in the review.

ID	Date Country	Sample	Method	N (%)	Self-/Non self-	Quality
Tangney, 1987 [32]	n.s US	Researchers in a “highly ranked American university”.	Distributed within department	245 (22)	n	1
Lock, 1988 [29]	1988 UK	Professors of medicine or surgery, other academics, doctors, research managers, editors of medical journals non-randomly contacted by the author	Mailed+pre-paid return	79 (98.7)	n	0
Simmons, 1991 [54]	1989 US	Active members of the Society of University Surgeons	n.s.	202 (82)	n	0
Kalichman, 1992 [35]	1990 US	Research trainees in the clinical and basic biomedical sciences at the University of California, San Diego	Distributed through the department	549 (27)	s+n	1
Swazey, 1993 [53]	1990 US	Doctoral students and faculty, from 99 of the largest graduate departments in chemistry, civil engineering, microbiology and sociology	Mailed+prepaid return+postcard to confirm response	2620 (65.5)	n	1
Glick, 1993 [55]	1992 US	Biotechnology companies' executives known by the author	Administered orally, on the phone	15* (n.s)	n	0
Greenberg, 1994 [20]	1991 US	Members of the Society for Risk Analysis, Association of Environmental and Resource Economists, American Industrial Hygiene Association	Mailed	478 (32)	n	1
Glick, 1994 [30]	1993 US	Attendees at the Third Conference on Research Policies and Quality Assurance	Handed out, personally returned by respondents on the same	36 (34)	n	1
Eastwood, 1996 [56]	1993 US	All postdoctoral fellows registered with the Office of Research Affairs of the University of California, San Francisco	Mailed+follow-up letter	324 (32.8)	s+n	1
Bebeau, 1996 [33]	1995 US	Program chairs and officers of the American Association for Dental Research	Mailed+prepaid return+postcard to confirm response	76 (78)	n	1
Rankin, 1997 [57]	1995 US	Research coordinators or directors of master's and doctoral nursing programs	Mailed	88 (43)	n	1
May, 1998 [34]	1997 UK	Randomly selected authors of papers published in the past 3 years on addiction-related subjects	Mailed	36 (51)	n	1
Ranstam, 2000 [46]	1998 Various	Members of the International Society of Clinical Biostatistics	Mailed+online electronic version	163 (37)	n	1
List, 2001 [28]	1998 US	Participants to the January 1998 meetings of the American Economic Association	Hand-delivered, Direct Response+Random Response method, drop box for returning responses	94 (23.5)	s	1
Geggie, 2001 [58]	2000 UK	Medical consultants appointed between Jan 1995 and Jan 2000 working in 7 hospital trusts in the Mersey region	Mailed+pre-paid return	194 (63.6)	s+n	1
Meyer, 2004 [59]	n.s US	Members of editorial boards of American Accounting Association journals, and participants at the 1998, 1999, and 2000 American Accounting Association New Faculty Consortia	Email asking to reply if unwilling to participate, mailed+pre-paid return	176 (48.5)	n	1
Martinson, 2005 [19]	2002 US	Researchers funded by the National Institutes of Health	Mailed, pre-paid return, 2$	3247 (47.2)	s	1
Henry, 2005 [60]	2002 Australia	Medical specialists, from the 2002 edition of the Medical directory of Australia, involved in pharmaceutical industry-sponsored research	Mailed	338* (n.a.)	s	1
Gardner, 2005 [27]	2002 Various	Authors of pharmaceutical clinical trials published in the Cochrane Database of Systematic Reviews, equally selected between first, middle and last author.	Mailed+10$ check+second survey to non-respondents+follow-up call or email	322 (64)	s+n	1
Kattenbraker 2007 [61]	2005 US	Health education professors at every rank, teaching at 94 institution of higher education	Email+web-based survey+follow up email+final reminder	153 (25.8)	n	1
Titus, 2008 [31]	2005 US	Researchers funded by the National Institutes of Health, one per department	Pre-notification+mailed+reminder postcard+additional survey packet+follow-up letter	2212 (52)	n	1

Abbreviations: “Date” is the year when the survey was actually conducted, “N” is the number of respondents who returned the questionnaire, “%” is the response rate of the survey.

* Number of respondents who ad engaged in industry-sponsored research in the previous 12 months, out of a total sample of 2253, with 39% response rate.

### Quantitative data analysis

#### Scientists admitting misconduct

**Figure 2:**
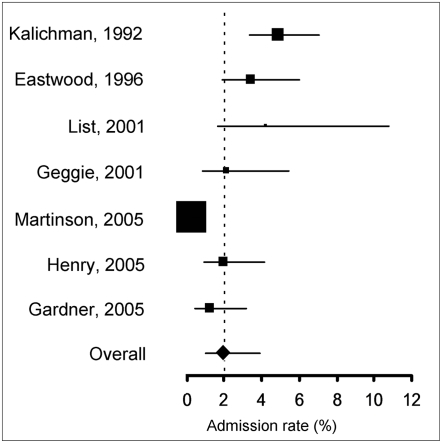
Forrest plot of admission rates of data fabrication, falsification and alteration in self reports. Area of squares represents sample size, horizontal lines are 95% confidence interval, diamond and vertical dotted line show the pooled weighted estimate.

When explicitly asked if they ever fabricated or falsified research data, or if they altered or modified results to improve the outcome (see Table S2, questions 1, 4, 6, 8, 10, 17, 26), between 0.3% and 4.9% of scientists replied affirmatively (N = 7, crude unweighted mean: 2.59%, 95%CI = 1.06–4.13). Meta-analysis yielded a pooled weighted estimate of 1.97% (95%CI: 0.86–4.45), with significant heterogeneity (Cochran's Q = 61.7777, df = 6, P<0.0001) (Figure 2). If only questions explicitly using the words “fabrication” or “falsification” were included (Table S2, questions 3, 6, 10, 26), the pooled weighted estimate was 1.06% (N = 4, 95%CI: 0.31–3.51)

**Figure 3:**
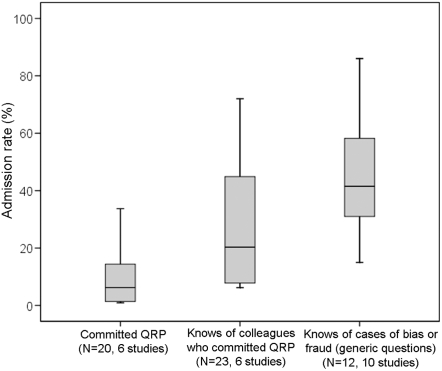
Admission rates of Questionable Research Practices (QRP) in self- and non-self-reports. N indicates the number of survey questions. Boxplots show median and interquartiles.

Other questionable practices were admitted by up to 33.7% of respondents (Table S2) (Figure 3, N = 20 (six studies), crude unweighted mean: 9.54%, 95%CI = 5.15–13.94).

Consistently across studies, scientists admitted more frequently to have “modified research results” to improve the outcome than to have reported results they “knew to be untrue” (Inverse Variance Weighted Oneway ANOVA Q(1,4) = 14.8627, P = 0.011)

In discussing limitations of results, two studies [19], [27] suggested that their results were very conservative with respect to the actual occurrence of misconduct, while the other studies made no clear statement. Non-response bias was recognized as a limitation by most surveys. One study employed a Random-Response technique on part of its sample to control for non-response bias, and found no evidence for it [28] (see Discussion for further details).

#### Scientists observing misconduct

**Figure 4:**
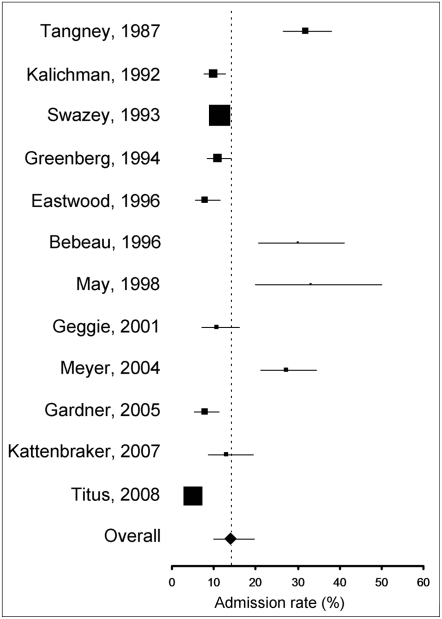
Forrest plot of admission rates of data fabrication, falsification and alteration in non-self reports. Area of squares represents sample size, horizontal lines are 95% confidence interval, diamond and vertical dotted line show the pooled weighted estimate.

When asked if they had personal knowledge of a colleague who fabricated or falsified research data, or who altered or modified research data (Table S3, questions, 1, 6, 7, 10, 20, 21, 29, 32, 34, 37, 45, 54) between 5.2% and 33.3% of respondents replied affirmatively (N = 12, crude unweighted mean: 16.66%, 95%CI = 9.91–23.40). Meta-analysis yielded a pooled weighted estimate of 14.12% (95% CI: 9.91–19.72) (Figure 4). If only questions explicitly using the words “fabrication” or “falsification” were included (Table S3, questions 1, 6, 7, 10, 17, 21, 29, 32, 37, 45, 54), the pooled weighted estimate was 12.34% (N = 11, 95%CI: 8.43–17.71)

Between 6.2% and 72% of respondents had knowledge of various questionable research practices (Table S3) (Figure 3, N = 23 (6 studies), crude unweighted mean: 28.53%, 95%CI = 18.85–38.2). When surveys asked about more generic questions (e.g. “do you have knowledge of any cases of fraud?” [29], [30]) or defined misconduct in more comprehensive ways (e.g. “experimental deficiencies, reporting deficiencies, misrepresentation of data, falsification of data” [30]) between 12% and 92% replied affirmatively (Table S3) (N = 10 (seven studies), crude unweighted mean: 46.24, 95%CI = 16.53–75.95).

In discussing their results, three studies [27], [29], [31] considered them to be conservative, four [30], [32], [33], [34] suggested that they overestimated the actual occurrence of misconduct, and the remaining 13 made no clear statement.

#### Scientists reporting misconduct

Five of the included studies asked respondents what they had done to correct or prevent the act of misconduct they had witnessed. Around half of the alleged cases of misconduct had any action taken against them (Table 2). No study asked if these actions had the expected outcome. One survey [27] found that 29% of the cases of misconduct known by respondents were never discovered.

**Table 2:** Actions taken against misconduct.

ID	N cases	Action taken	%
Tangney, 1987 [32]	78	Took some action to verify their suspicions of fraud or to remedy the situation	46
Rankin, 1997 [57]	31 [ffp]	In alleged cases of scientific misconduct a disciplinary action was taken by the dean	32.4
		Some authority was involved in a disciplinary action	20.5
Ranstam, 2000 [46]	49	I interfered to prevent it from happening	28.6
		I reported it to a relevant person or organization	22.4
Kattenbraker, 2007 [61]	33	Confronted individual	55.5
		Reported to supervisor	36.4
		Reported to Institutional Review Board	12.1
		Discussed with colleagues	36.4
Titus, 2008 [31]	115 [ffp]	The suspected misconduct was reported by the survey respondent	24.4
		The suspected misconduct was reported by someone else	33.3

Abbreviations: “N cases” is the total number of cases of misconduct observed by respondents, [ffp] indicates that the number includes cases of plagiarism, “%” is the percentage of cases that had the specified action taken against them. All responses are mutually exclusive except in Kattenbraker 2007.

#### Factors influencing responses

Methodological differences between studies explained a large portion of the variance among effect sizes (N = 15, one outcome per study, Table 3). Lower percentages of misconduct were reported in self reports, in surveys using the words “falsification” or “fabrication”, and in mailed surveys. Mailed surveys had also higher response rates than handed-out surveys (Mean: 26.63%±2.67SE and 48.53%±4.02SE respectively, t-test: t = −2.812, df = 16, P = 0.013), while no difference in response rates was observed between self- and non-self-reports (Mean: 42.44±6.24SE and 44.44±5.1SE respectively, t = −0.246, P = 0.809) and between surveys using or not “fabrication or falsification” (Mean: 42.98%±6.0SE and 44.51±4.76SE respectively, t = −0.19, P = 0.85). Excluding all surveys that were not mailed, were not self-reports and that did not use the words “falsification” or “fabrication” yielded a maximally conservative pooled weighted estimate of 0.64% (N = 3, 95%CI: 0.25–1.63).

**Table 3:** Inverse variance-weighted regression on admission rates.

	Variable	B±SE	P	Stand. Coeff.	Model R^2^
Base Model	Constant	−4.53±0.81	<0.0001	0	0.82
	Self-/Non-self	−3.02±0.38	<0.0001	−1.04	
	Mailed/Handed	−1.17±0.4	0.0032	−0.33	
	“Fabricated, Falsified”/“Modified”	−1.02±0.39	0.0086	−0.34	
Candidate co-variables	Year	−0.03±0.03	0.3	−0.14	0.83
	USA/other	−0.71±0.4	0.08	−0.2	0.85
	Researcher/other	−0.33±0.33	0.32	−0.11	0.83
	Biomedical/other	0.17±0.39	0.66	0.06	0.82
	Medical/other	0.85±0.28	0.0022	0.29	0.89
	Social Sc./other	−0.03±0.37	0.94	−0.01	0.82

The table shows model parameters of an initial model including three methodological factors (top four rows) and the parameter values for each sample characteristic, entered one at a time in the basic model. All variables are binary. Regression slopes measure the change in admission rates when respondents fall in the first category.

When the three methodological factors above where controlled for, a significant effect was found for surveys targeted at medical and clinical researchers, who reported higher percentages of misconduct than respondents in biomedical research and other fields (Table 3). The effect of this parameter would remain significant if Bonferroni-corrected for multiple comparisons. If self- and non-self- reports were tested separately for the effect of study characteristics (one characteristic at a time), a significant effect was found only in self-reports for year when survey was conducted (k = 7, b = −0.1425±0.0519, P = 0.006) and a nearly significant effect was found again in self-reports for survey delivery method (k = 7, b = −1.2496±0.6382, P = 0.0502)

### Sensitivity analysis

**Figure 5:**
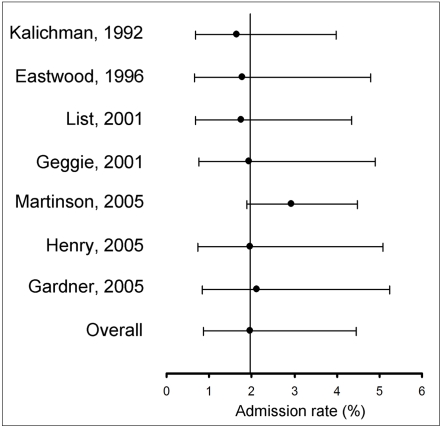
Sensitivity analysis of admission rates of data fabrication, falsification and alteration in self reports. Plots show the weighted pooled estimate and 95% confidence interval obtained when the corresponding study was left out of the analysis.

**Figure 6:**
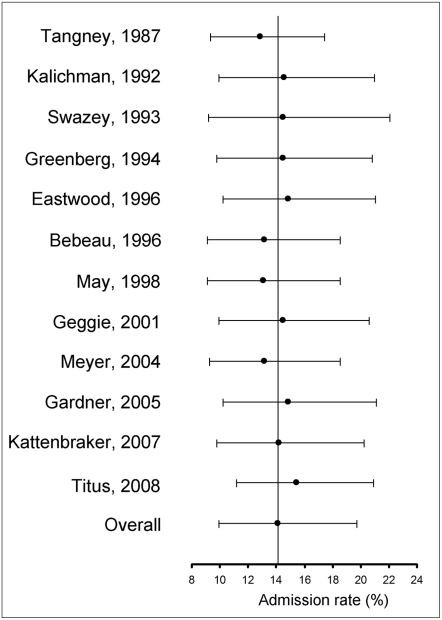
Sensitivity analysis of admission rates of data fabrication, falsification and alteration in non-self reports. Plots show the weighted pooled estimate obtained when the corresponding study was left out of the analysis.

Self-report admission rates varied between 1.65% -following the removal of Kalichman and Friedman (1992) [35]- and 2.93% -following the removal of Martinson et al. (2005) [19] (Figure 5). Reports on colleagues' misconduct varied between 12.85% (when Tangney (1987) [32] was removed) and 15.41% (when Titus et al. (2008) [31] was removed (Figure 6). Weighted pooled estimates on non-logit-trasformed data yielded self- and non-self- admission rates of 2.33% (95%CI 0.94–3.73%) and 14.48% (95%CI: 11.14–17.81%) respectively, showing that the results are robust and conservative.

Results of the regression analysis were robust to the leave-one-study-out test: the four significant variables remained statistically significant when anyone of the studies was excluded (Table S4). The largest portion of variance was explained when Titus et al. (2008) [31] was removed (R^2^ = 0.9202). Meta-regression on non-transformed data showed similar trends to that on transformed data for all four parameters, but only two parameters remained statistically significant (self-/non-self- and delivery method, P<0.0001 and p = 0.0083 respectively), and the overall portion of variance explained by the model was lower (R^2^ = 0.6904).

## Discussion

This is the first meta-analysis of surveys asking scientists about their experiences of misconduct. It found that, on average, about 2% of scientists admitted to have fabricated, falsified or modified data or results at least once –a serious form of misconduct my any standard [10], [36], [37]– and up to one third admitted a variety of other questionable research practices including “dropping data points based on a gut feeling”, and “changing the design, methodology or results of a study in response to pressures from a funding source”. In surveys asking about the behaviour of colleagues, fabrication, falsification and modification had been observed, on average, by over 14% of respondents, and other questionable practices by up to 72%. Over the years, the rate of admissions declined significantly in self-reports, but not in non-self-reports.

A large portion of the between-studies variance in effect size was explained by three basic methodological factors: whether the survey asked about self or not, whether it was mailed or handed out to respondents, and whether it explicitly used the words “fabrication” and “falsification”. Once these factors were controlled for, surveys conducted among clinical, medical and pharmacological researchers appeared to yield higher rates of misconduct than surveys in other fields or in mixed samples.

All the above results were robust with respect to inclusion or exclusion of any particular study, with perhaps one exception: Martinson et al. (2005) [19], which is one of the largest and most frequently cited surveys on misconduct published to date. This study appears to be rather conservative, because without it the pooled average frequency with which scientists admit they have committed misconduct would jump to nearly 3%.

How reliable are these numbers? And what can they tell us on the actual frequency of research misconduct? Below it will be argued that, while surveys asking about colleagues are hard to interpret conclusively, self-reports systematically underestimate the real frequency of scientific misconduct. Therefore, it can be safely concluded that data fabrication and falsification –let alone other questionable research practices- are more prevalent than most previous estimates have suggested.

The procedure adopted to standardize data in the review clearly has limitations that affect the interpretation of results. In particular, the percentage of respondents that recall at least one incident of misconduct is a very rough measure of the frequency of misconduct, because some of the respondents might have committed several frauds, but others might have “sinned” only once. In this latter case, the frequencies reported in surveys would tend to overestimate the prevalence of biased or falsified data in the literature. The history of science, however, shows that those responsible of misconduct have usually committed it more than once [38], [39], so the latter case might not be as likely as the former. In any case, many of the included studies asked to recall at least one incident, so this limitation is intrinsic to large part of the raw data.

The distinction made in this review between “fabrication, falsification and alteration” of results and QRP is somewhat arbitrary. Not all alterations of data are acts of falsification, while “dropping data points based on a gut feeling” or “failing to publish data that contradicts one's previous research” (e.g. [19]) might often be. As explained in the introduction, any boundary defining misconduct will be arbitrary, but intention to deceive is the key aspect. Scientists who answered “yes” to questions asking if they ever fabricated or falsified data are clearly admitting their intention to misrepresent results. Questions about altering and modifying data “to improve the outcome” might be more ambiguously interpreted, which might explain why these questions yield higher admission rates. However, even if we limited the meta-analysis to the most restrictive types of questions in self-reports, we would still have an average admission rate above 1%, which is higher than previous estimates (e.g. [11]).

The accuracy of self-reports on scientific misconduct might be biased by the effect of social expectations. In self-reports on criminal behaviour, social expectations make many respondents less likely to admit a crime they committed (typically, females and older people) and make others likely to report a crime they have not really committed (typically, young males) [40]. In the case of scientists, however, social expectations should always lead to underreporting, because a reputation of honesty and objectivity is fundamental in any stage of a scientific career. Anyone who has ever falsified research is probably unwilling to reveal it and/or to respond to the survey despite all guarantees of anonymity [41]. The opposite (scientists admitting misconduct they didn't do) appears very unlikely. Indeed, there seems to be a large discrepancy between what researchers are willing to do and what they admit in a survey. In a sample of postdoctoral fellows at the University of California San Francisco, USA, only 3.4% said they had modified data in the past, but 17% said they were “willing to select or omit data to improve their results” [42]. Among research trainees in biomedical sciences at the University of California San Diego, 4.9% said they had modified research results in the past, but 81% were “willing to select, omit or fabricate data to win a grant or publish a paper” [35].

Mailed surveys yielded lower frequencies of misconduct than handed out surveys. Which of the two is more accurate? Mailed surveys were often combined with follow-up letters and other means of encouraging responses, which ensured higher response rates. However, the accuracy of responses to sensitive questions is often independent of response rates, and depends strongly on respondents' perception of anonymity and confidentiality [43], [44]. Questionnaires that are handed to, and returned directly by respondents might better entrust anonymity than surveys that need to be mailed or emailed. Therefore, we cannot rule out the possibility that handed out surveys are more accurate despite the lower response rates. This latter interpretation would be supported by one of the included studies: a handed out survey that attempted to measure non-response bias using a Random-Response (RR) technique on part of its sample [28]. Differently from the usual Direct Response technique, in RR, respondents toss coins to determine whether they will respond to the question or just mark “yes”. This still allows admission rates to be calculated, yet it guarantees full anonymity to respondents because no one can tell whether an individual respondent answered “yes” to the question or because of chance. Contrary to author's expectations, response and admission rates were not higher with RR compared to DR, suggesting that in this handed out survey non-response bias was absent.

The effect of social expectations in surveys asking about colleagues is less clear, and could depend on the particular interests of respondents. In general, scientists might tend to protect the reputation of their field, by minimizing their knowledge of misconduct [27]. On the other hand, certain categories of respondents (e.g. participants at a Conference on Research Policies and Quality Assurance [30]) might have particular experience with misconduct and might be very motivated to report it.

Surveys on colleagues' behaviour might tend to inflate estimates of misconduct also because the same incident might be reported by many respondents. One study controlled for this factor by asking only one researcher per department to recall cases that he had observed in that department in the past three years [31]. It found that falsification and fabrication had been observed by 5.2% of respondents, which is lower than all previous non-self reports. However, since one individual will not be aware of all cases occurring around him/her, this is a conservative estimate [31]. In the sensitivity analysis run on the regression model, exclusion of this study caused the single largest increase in explained variance, which further suggests that findings of this study are unusual.

Another critical factor in interpreting survey results is the respondents' perception of what does and does not constitute research misconduct. As mentioned before, scientists were less likely to reply affirmatively to questions using the words “fabrication” and “falsification” rather than “alteration” or “modification”. Moreover, three surveys found that scientists admitted more frequently to have “modified” or “altered” research to “improve the outcome” than to have reported results they “knew to be untrue”. In other words, many did not think that the data they “improved” were falsified. To some extent, they were arguably right. But the fuzzy boundary between removing noise from results and biasing them towards a desired outcome might be unknowingly crossed by many researchers [10], [14], [45]. In a sample of biostatisticians, who are particularly well trained to see this boundary, more than half said they had personally witnessed false or deceptive research in the preceding 10 years [46].

The grey area between licit, questionable, and fraudulent practices is fertile ground for the “Mohammed Ali effect”, in which people perceive themselves as more honest than their peers. This effect was empirically proven in academic economists [28] and in a large sample of biomedical researchers (in a survey assessing their adherence to Mertonian norms [47]), and may help to explain the lower frequency with which misconduct is admitted in self-reports: researchers might be overindulgent with their behaviour and overzealous in judging their colleagues. In support of this, one study found that 24% of cases observed by respondents did not meet the US federal definition of research misconduct [31].

The decrease in admission rates observed over the years in self-reports but not in non-self-reports could be explained by a combination of the Mohammed Ali effect and social expectations. The level and quality of research and training in scientific integrity has expanded in the last decades, raising awareness among scientists and the public [11]. However, there is little evidence that researchers trained in recognizing and dealing with scientific misconduct have a lower propensity to commit it [47], [48], [49]. Therefore, these trends might suggest that scientists are no less likely to commit misconduct or to report what they see their colleagues doing, but have become less likely to admit it for themselves.

Once methodological differences were controlled for, cross-study comparisons indicated that samples drawn exclusively from medical (including clinical and pharmacological) research reported misconduct more frequently than respondents in other fields or in mixed samples. To the author's knowledge, this is the first cross-disciplinary evidence of this kind, and it suggests that misconduct in clinical, pharmacological and medical research is more widespread than in other fields. This would support growing fears that the large financial interests that often drive medical research are severely biasing it [50], [51], [52]. However, as all survey-based data, this finding is open to the alternative interpretation that respondents in the medical profession are simply more aware of the problem and more willing to report it. This could indeed be the case, because medical research is a preferred target of research and training programs in scientific integrity, and because the severe social and legal consequences of misconduct in medical research might motivate respondents to report it. However, the effect of this parameter was not robust to one of the sensitivity analyses, so it would need to be confirmed by independent studies before being conclusively accepted.

The lack of statistical significance for the effect of country, professional position and other sample characteristics is not strong evidence against their relevance, because the high between-study variance caused by methodological factors limited the power of the analysis (the regression had to control for three methodological factors before testing any other effect). However, it suggests that such differences need to be explored at the study level, with large surveys designed specifically to compare groups. A few of the included studies had done so and found, for example, that admission rates tend to be higher in males compared to females [42] and in mid-career compared to early career scientists [19], and that they tend to differ between disciplines [41], [53]. If more studies attempted to replicate these results, possibly using standardized methodologies, then a meta-analysis could reveal important correlates of scientific misconduct.

In conclusion, several surveys asking scientists about misconduct have been conducted to date, and the differences in their results are largely due to differences in methods. Only by controlling for these latter can the effects of country, discipline, and other demographic characteristics be studied in detail. Therefore, there appears to be little scope for conducting more small descriptive surveys, unless they adopted standard methodologies. On the other hand, there is ample scope for surveys aimed at identifying sociological factors associated with scientific misconduct. Overall, admission rates are consistent with the highest estimates of misconduct obtained using other sources of data, in particular FDA data audits [11], [18]. However, it is likely that, if on average 2% of scientists admit to have falsified research at least once and up to 34% admit other questionable research practices, the actual frequencies of misconduct could be higher than this.

## Supporting Information

Table S1 Studies excluded from the review.
(0.14 MB DOC)

Table S2 Self-report questions included in review, and responses.
(0.07 MB DOC)

Table S3 Non-self report questions included in the review, and responses.
(0.11 MB DOC)

Table S4 Sensitivity analysis for meta-regression model.
(0.07 MB DOC)
