# Supplementary material for: How Many Scientists Fabricate and Falsify Research? A Systematic Review and Meta-Analysis of Survey Data
Source: PLoS One. 2009 May 29;4(5):e5738. doi: 10.1371/journal.pone.0005738 (PMC2685008; doi:10.1371/journal.pone.0005738)
Supplement: Table S1 — Studies excluded from the review. (0.14 MB DOC) [file pone.0005738.s001.doc]

Table S1: Studies excluded from the review.

| **Study** | **Reasons for exclusion** |
| --- | --- |
| (Aggarwal, Bates et al. 2002) | Sample consists of students |
| (Alt-White and Pranulis 2006) | No data on the occurrence of misconduct |
| (Anderson, Louis et al. 1994) | No data on the occurrence of misconduct |
| (Andrews, Smith et al. 2007) | Misconduct is only considered in relation to school activities, not of publishable research |
| (Atkinson, Gilleland et al. 2007) | No data on the occurrence of misconduct |
| (Bailey, Hasselback et al. 2001) | Does not clearly distinguish between self-report and opinion on the percentage of literature affected by misconduct |
| (Blumenthal, Campbell et al. 1997) | No data on the occurrence of misconduct |
| (Blumenthal, Campbell et al. 2006) | No data on the occurrence of misconduct |
| (Braxton and Bayer 1994) | No data on the occurrence of misconduct |
| (Braxton and Bayer 1996) | No data on the occurrence of misconduct |
| (Brimble and Stevenson-Clarke 2005) | Misconduct only considered in relation to school activities, not of publishable research |
| (Brown and Kalichman 1998) | No data on the occurrence of misconduct |
| Campbell and Bendavid 2003) | No data on the occurrence of misconduct |
| (Campbell, Clarridge et al. 2002) | No data on the occurrence of misconduct |
| (Campbell, Weissman et al. 2000; Campbell and Bendavid 2003) | No data on the occurrence of misconduct |
| (Castleberry, French et al. 1993) | No data on the occurrence of misconduct |
| (Cossette 2004) | In the questions relevant to this study, does not distinguish between different forms of misconduct (e.g. plagiarism from falsification and fabrication) |
| (Davis, Wester et al. 2008) | No data on the occurrence of misconduct |
| (Heitman, Olsen et al. 2007) | No data on the occurrence of misconduct |
| (Jacobsen and Hals 1995) | Does not distinguish between different forms of misconduct (e.g. plagiarism from falsification and fabrication) |
| (Ketefian and Lenz 1995) | No data on the occurrence of misconduct |
| (Korenman and Viosca 1993) | Does not distinguish between different forms of misconduct (e.g. plagiarism from falsification and fabrication) |
| (Louis, Anderson et al. 1995) | Data is presented in mean and St. Dev. |
| (Lynöe, Jacobsson et al. 1999) | No data on the occurrence of misconduct |
| (Marsden, Carroll et al. 2005) | Sample consists only of students, and data is presented in means and St. Dev. |
| (Martinson, Anderson et al. 2006) | Same data used in |
| (Mason 1990) | No data on the occurrence of misconduct |
| (McKay, Kidwell et al. 2007) | No data on the occurrence of misconduct |
| (Mumford, Devenport et al. 2006) | No data on the occurrence of misconduct |
| (Mumford, Murphy et al. 2007) | No data on the occurrence of misconduct |
| (Mundt 2007) | No data on the occurrence of misconduct |
| (Pérez-Tejada and Macías-Ordóñez 2003) | No data on the occurrence of misconduct |
| (Plemmons, Brody et al. 2006) | No data on the occurrence of misconduct |
| (Pryor, Habermann et al. 2007) | In the questions relevant to this study, does not distinguish between different forms of misconduct (e.g. plagiarism from falsification and fabrication) |
| (Rennie and Crosby 2001) | Sample consists of students |
| (Rhoades 2002) | No original data |
| (Rhoades 2003) | No data on the occurrence of misconduct |
| (Riis 1999) | No original data |
| (Saint James-Roberts 1976) | Open survey, with sample largely unspecified and clearly not composed exclusively of researchers or even scientists |
| (Semerci 2006) | Misconduct is only considered in relation to school activities, not of publishable research |
| (Sempolski 1986) | No data on the occurrence of misconduct |
| (Sheehan, Sheehan et al. 1990) | Sample consists only of students |
| (Tang and Chamberlain 1997) | No data on the occurrence of misconduct |
| (Turrens 2005) | No data on the occurrence of misconduct |
| (Vogeli, Yucel et al. 2006) | No data on the occurrence of misconduct |
| (Wenger, Korenman et al. 1999) | No data on the occurrence of misconduct |
| (Wester, Willse et al. 2008) | No data on the occurrence of misconduct |
| (Zubin, Collins et al. 2006) | Sample consists only of students |

Aggarwal, R., I. Bates, et al. (2002). "A study of academic dishonesty among students at two pharmacy schools." The Pharmaceutical Journal **269**: 529-532.

Alt-White, A. C. and M. F. Pranulis (2006). "Addressing nurses' ethical concerns about research in critical care settings." Nurs Adm Q **30**(1): 67-75.

Anderson, M. S., K. S. Louis, et al. (1994). "Disciplinary and departmental effects on observations of faculty and graduate student misconduct." The Journal of Higher Education **65**(3): 331-350.

Andrews, K. G., L. A. Smith, et al. (2007). "Faculty and student perceptions of academic integrity at U.S. and Canadian dental schools." Journal of Dental Education **71**(8): 1027-1039.

Atkinson, T. N., D. S. Gilleland, et al. (2007). "The Research Environment Norm Inventory (RENI): a study of integrity in research administrative systems." Account Res **14**(2): 93-119.

Bailey, C. D., J. R. Hasselback, et al. (2001). "Research misconduct in accounting literature: a survey of the most prolific researchers' actions and beliefs." ABACUS **37**(1): 26-54.

Blumenthal, D., E. G. Campbell, et al. (1997). "Withholding research results in academic life science - evidence from a national survey of faculty." Jama-Journal of the American Medical Association **277**(15): 1224-1228.

Blumenthal, D., E. G. Campbell, et al. (2006). "Data withholding in genetics and the other life sciences: prevalences and predictors." Academic Medicine **81**(2): 137-145.

Braxton, J. M. and A. E. Bayer (1994). "Perceptions of research misconduct and an analysis of their correlates" Journal of Higher Education **65**(3): 351-372.

Braxton, J. M. and A. E. Bayer (1996). "Personal experiences of research misconduct and the response of individual academic scientists." Science, Technology & Human Values **21**(2): 198-213.

Brimble, M. and P. Stevenson-Clarke (2005). "Perceptions of the prevalence and seriousness of academic dishonesty in Australian universities." The Australian Educational Researcher **32**(3): 19-44.

Brown, S. and M. W. Kalichman (1998). "Effects of training in the responsible conduct of research: a survey of graduate students in experimental sciences." Science and Engineering Ethics **4**(4): 487-498.

Campbell, E. G. and E. Bendavid (2003). "Data-sharing and data-withholding in genetics and the life sciences: results of a national survey of technology transfer officers." J Health Care Law Policy **6**(2): 241-55.

Campbell, E. G., B. R. Clarridge, et al. (2002). "Data withholding in academic genetics - evidence from a national survey." Jama-Journal of the American Medical Association **287**(4): 473-480.

Campbell, E. G., J. S. Weissman, et al. (2000). "Data withholding in academic medicine: characteristics of faculty denied access to research results and biomaterials." Research Policy **29**(2): 303-312.

Castleberry, S. B., W. French, et al. (1993). "The ethical framework of advertising and marketing research practitionser- a moral development perspective" Journal of Advertising **22**(2): 39-46.

Cossette, P. (2004). "Research integrity: an exploratory survey of administrative science faculties." Journal of Business Ethics **49**(3): 213-234.

Davis, M. S., K. L. Wester, et al. (2008). "Narcissism, entitlement, and questionable research practices in counseling: a pilot study." Journal of Counseling and Development **86**(2): 200-210.

Heitman, E., C. H. Olsen, et al. (2007). "New graduate students' baseline knowledge of the responsible conduct of research." Academic Medicine **82**(9): 838-845.

Jacobsen, G. and A. Hals (1995). "Medical investigators views about ethics and fraud in medical research" Journal of the Royal College of Physicians of London **29**(5): 405-409.

Ketefian, S. and E. R. Lenz (1995). "Promoting scientific integrity in nursing research 2. Strategies." Journal of Professional Nursing **11**(5): 263-269.

Korenman, S. G. and S. P. Viosca (1993). "Scientist and trainee experience in research integrity." Clinical Research **41**(2): A289-A289.

Louis, K. S., M. S. Anderson, et al. (1995). "Academic misconduct and values: the department's influence." The Review of Higher Education **18**(4): 393-422.

Lynöe, N., L. Jacobsson, et al. (1999). "Fraud, misconduct or normal science in medical research - an empirical study of demarcation." Journal of Medical Ethics **25**: 501-506.

Marsden, H., M. Carroll, et al. (2005). "Who cheats at university? A self-report study of dishonest academic behaviours in a sample of Australian university students." Australian Journal of Psychology **57**(1): 1-10.

Martinson, B. C., M. S. Anderson, et al. (2006). "Scientists’ perceptions of organizational justice and self-reported misbehaviours." Journal of Empirical Research on Human Research Ethics **1**(1): 51-66.

Mason, B. J. (1990). "Perceived conduct and professional ethics among marketing faculty." Journal of the Academy of Marketing Science **18**(3): 185-197.

McKay, R. B., L. A. Kidwell, et al. (2007). "Faculty ethics from the perspective of college of business administrators." Journal of Legal, Ethical and Regulatory Issues **10**(1): 105-124.

Mumford, M. D., L. D. Devenport, et al. (2006). "Validation of ethical decision making measures: evidence for a new set of measures." Ethics & Behavior **16**(4): 319-345.

Mumford, M. D., S. T. Murphy, et al. (2007). "Environmental influences on ethical decision making: climate and environmental predictors of research integrity." Ethics & Behavior **17**(4): 337-366.

Mundt, L. A. (2007). Perceptions of scientific misconduct among graduate allied health students relative to ethics education and gender, University of Phoenix. Doctoral dissertation.

Pérez-Tejada, C. D. and R. Macías-Ordóñez (2003). “El que no transa no avanza”: la ciencia mexicana en el espejo. El Papel de la Ética en la Investigación Científica y la Educación Superior. M. A. A. Birke, Academia Mexicana de Ciencias, México D.F.**:** 133-153.

Plemmons, D. K., S. A. Brody, et al. (2006). "Student perceptions of the effectiveness of education in the responsible conduct of research." Science and Engineering Ethics **12**(3): 571-582.

Pryor, E. R., B. Habermann, et al. (2007). "Scientific misconduct from the perspective of research coordinators: a national survey." Journal of Medical Ethics **33**(6): 365-369.

Rennie, S. C. and J. R. Crosby (2001). "Are "tomorrow's doctors" honest? Questionnaire study exploring medical studnets' attitudes and reported behaviour on academic misconduct." British Medical Journal **322**: 274-275.

Rhoades, L. J. (2002). "Beyond conflict of interest: the responsible conduct of research." Science and Engineering Ethics **8**(3): 459-468.

Rhoades, L. J. (2003). Survey of research integrity measures utilized in biomedical research laboratories. Final report. A. I. f. Research, Office of Research Integrity, DHHS.

Riis, P. (1999). "Misconduct in clinical research - the Scandinavian experience and actions for prevention." Acta Oncologica **38**(1): 89-92.

Saint James-Roberts, I. (1976). "Cheating in science." New Scientist **72**(1028): 466-469.

Semerci, C. (2006). "The opinions of medicine faculty students regarding cheating in relation to kohlberg's moral development concept." Social Behavior and Personality **34**(1): 41-49.

Sempolski, N. J. (1986). A study of social control in science: responses of organizations to reports of misconduct Department of Anthropology. Lawrence, KS University of Kansas. Doctoral dissertation.

Sheehan, K. H., D. V. Sheehan, et al. (1990). "A pilot study of medical student "abuse": student perceptions of mistreatment and misconduct in medical school." Jama-Journal of the American Medical Association **263**: 533-537.

Tang, T. L. P. and M. Chamberlain (1997). "Attitudes toward research and teaching - differences between administrators and faculty members." Journal of Higher Education **68**(2): 212-&.

Turrens, J. F. (2005). "Teaching research integrity and bioethics to science undergraduates." Cell Biol Educ **4**(4): 330-4.

Vogeli, C., R. Yucel, et al. (2006). "Data withholding and the next generation of scientists: results of a national survey." Academic Medicine **81**(2): 128-136.

Wenger, N. S., S. G. Korenman, et al. (1999). "Reporting unethical research behavior." Evaluation Review **23**(5): 553-570.

Wester, K. L., J. T. Willse, et al. (2008). "Responsible conduct of research measure: initial development and pilot study." Account Res **15**(2): 87-104.

Zubin, A., D. Collins, et al. (2006). "Influence of attitudes toward curriculum on dishonest academic behaviour." American Journal of Pharmaceutical Education **70**(3): 1-9.
