# Supplementary material for: How Many Scientists Fabricate and Falsify Research? A Systematic Review and Meta-Analysis of Survey Data
Source: PLoS One. 2009 May 29;4(5):e5738. doi: 10.1371/journal.pone.0005738 (PMC2685008; doi:10.1371/journal.pone.0005738)
Supplement: Table S2 — Self-report questions included in review, and responses. (0.07 MB DOC) [file pone.0005738.s002.doc]

Table S2. Self-report questions included in review, and responses.

| **Num.** | **ID** | **Question** | **X (behavior)** | **% yes** |
| --- | --- | --- | --- | --- |
| 1 | Kalichman,  1992 [1] | Since entering a college/university have you X | Modified research or experimental results to improve the outcome | 4.9 |
| 2 |  |  | Reported research or experimental results which you knew to be untrue | 2 |
| 3 | Eastwood, 1996 [2] | Since entering a college or university have you X | Fabricated data | 0.9 |
| 4 |  |  | Altered data to improve the outcome | 3.4 |
| 5 |  |  | Reported research or experimental results that you knew to be untrue | 1.2 |
| 6 | List,  2001 [3] | Have you ever X | Falsified research data | 4.26 |
| 7 |  |  |  | 4.49 |
| 8 | Geggie,  2001 [4] | Since entering medical school have you X | Modified research or experimental results to improve the outcome | 2.1 |
| 9 |  |  | Reported research or experimental results which you knew to be untrue | 1 |
| 10 | Martinson,  2005 [5] | Have you engaged in X during the past three years | Falsifying or "cooking" research data | 0.3 |
| 11 |  |  | Failing to present data that contradict one's own previous research | 6 |
| 12 |  |  | Overlooking others' use of flawed data or questionable interpretation of data | 12.5 |
| 13 |  |  | Changing the design, methodology or results of a study in response to pressure from a funding source | 15.5 |
| 14 |  |  | Withholding details of methodology or results in papers or proposals | 10.8 |
| 15 |  |  | Using inadequate or inappropriate research designs | 13.5 |
| 16 |  |  | Dropping observations or data points from analyses based on a gut feeling that they were inaccurate | 15.3 |
| 17 | Henry,  2005 [6] | n.s. | Alteration of patient data or statistics (excluding the normal processes of data editing) | 2* |
| 18 |  |  | Concealment of relevant findings | 5.3* |
| 19 |  |  | Failure to publish key findings | 12.1* |
| 20 |  |  | Premature termination of a study by a company | 33.7* |
| 21 |  |  | Major protocol changes while study in progress (excludes changes mandated by independent committees) | 5* |
| 22 |  |  | Editing of report to make drug appear better than was justified by the study results | 6.5* |
| 23 |  |  | First draft of a report written by pharmaceutical company or contract research organization | 29.6* |
| 24 |  |  | Delay in presentation or publication of key findings unrelated to data integrity | 16.3* |
| 25 | Gardner,  2005 [7] | Was there X in the target publication | Fabrication or misrepresentation | 0.9 |
| 26 |  | Have you participated in research involving X during the last 10 years | Fabricated or falsified data | 1.2 |
| 27 |  |  | Deleted data in an unjustified way | 0.9 |
| 28 |  |  | Deceptive or misleading report of design | 0.9 |
| 29 |  |  | Deceptive or misleading report of data | 1.2 |
| 30 |  |  | Seriously misleading interpretation of results | 1.6 |
| 31 |  |  | At least one of the above | 4.7 |

Abbreviations: “Num” = is a progressive number given to each separate question, for reference purposes., “%yes” is the number of respondents who replied affirmatively.

* percentages are calculated on the 338 respondents that had been engaged in industry-sponsored research in the previous 12 months.

1. Kalichman MW, Friedman PJ (1992) A pilot study of biomedical trainees' perceptions concerning research ethics. Academic Medicine 67: 769-775.

2.  Eastwood S, Derish P, Leash E, Ordway S (1996) Ethical issues in biomedical research: perceptions and practices of postdoctoral research fellows responding to a survey. Science and Engineering Ethics 2: 89-114.

3. List JA, et al. (2001) Academic economists behaving badly? A survey on three areas of unethical behavior. Economic Inquiry 39: 162-170.

4. Geggie D (2001) A survey of newly appointed consultants' attitudes towards research fraud. Journal of Medical Ethics 27: 344-346.

5. Martinson BC, Anderson MS, de Vries R (2005) Scientists behaving badly. Nature 435: 737-738.

6. Henry DA, Kerridge IH, Hill SR, McNeill PM, Doran E, et al. (2005) Medical specialists and pharmaceutical industry-sponsored research: a survey of the Australian experience. Medical Journal of Australia 182: 557-560.

7. Gardner W, Lidz CW, Hartwig KC (2005) Authors' reports about research integrity problems in clinical trials. Contemporary Clinical Trials 26: 244-251.
