# Supplementary material for: How Many Scientists Fabricate and Falsify Research? A Systematic Review and Meta-Analysis of Survey Data
Source: PLoS One. 2009 May 29;4(5):e5738. doi: 10.1371/journal.pone.0005738 (PMC2685008; doi:10.1371/journal.pone.0005738)
Supplement: Table S3 — Non-self report questions included in the review, and responses. (0.11 MB DOC) [file pone.0005738.s003.doc]

Table S3: Non-self report questions included in the review, and responses.

| **Num.** | **ID** | **Question** | **X (behavior)** | **% yes** |
| --- | --- | --- | --- | --- |
| 1 | Tangney,  1987 [1] | Have you at some time suspected a colleague in your field of X? | Falsifying data | 32 |
| 2 | Lock,  1988 [2] | Have knowledge of any cases of X | Fraud | 58.2 |
| 3 | Simmons,  1991 [3] | Have you strongly suspected X | Fraud that has not been investigated in your university | 15 |
| 4 |  | Are you aware of at least one incidence of X | Fraud that has been openly investigated in your institution | 40 |
| 5 |  |  | Fraud that has been investigated by quiet inquiry | 43 |
| 6 | Kalichman,  1992 [4] | Do you have first hand knowledge of scientists intentionally X for the purpose of publication | Altering or fabricating data | 10 |
| 7 | Swazey,  1993 [5] | In this program, have you observed or had other direct evidence of X | Falsifying or "cooking" research data | 11.4 |
| 8 |  |  | Failing to present data that contradict one's own previous research | 13.5 |
| 9 |  |  | Overlooking others' use of flawed data or questionable interpretation of data | 19.7 |
| 10 | Greenberg,  1994 [6] | How often [you] personally witnessed or had direct knowledge of X | Data fabrication | 11 |
| 11 |  |  | Biased research design | 28 |
| 12 |  |  | Data destruction | 9 |
| 13 | Glick,  1993 [7] | n.s. | Falsification of data or fabrication of experimental results; or experimental deficiencies; or reporting deficiencies; or misrepresentation of data | 60 |
| 14 | Glick,  1994 [8] | Have you ever suspected or determined that other researchers had been responsible for performing X | experimental deficiencies, reporting deficiencies, misrepresentation of data, falsification of data | 86 |
| 15 | Eastwood,  1996 [9] | Do you have firsthand knowledge of a scientist intentionally X | Fabricating data for a presentation | 4 |
| 16 |  |  | Fabricating data for a grant application | 3 |
| 17 |  |  | Fabricating data for a publication | 3 |
| 18 |  |  | Altering data for a presentation | 12 |
| 19 |  |  | Altering data for a grant application | 8 |
| 20 |  |  | Altering data for a publication | 8 |
| 21 | Bebeau,  1996 [10] | Indicate the number of IADR/AADR members you have observed/experienced exhibiting X within the last 5 years | Falsifying or "cooking" research data | 30 |
| 22 |  |  | Failing to present data that contradict one's own previous research | 51 |
| 23 |  |  | Overlooking others' use of flawed data or questionable interpretation of data | 72 |
| 24 |  |  | Failure to correct misinterpretation of data | 59 |
| 25 |  |  | Keeping research findings secret for several years | 30 |
| 26 |  |  | Failure to share data, methods and/or cultures that would enable others to re-examine/replicate or conduct further research | 44 |
| 27 |  |  | Failure to present negative results of corporate-sponsored research | 41 |
| 28 | Rankin,  1997 [11] | Are you aware that another faculty member had X | Cheated on a research project | 35.2 |
| 29 | May,  1998 [12] | In the preceding four years, have you direct personal knowledge of X | Misrepresentation or fabrication of data or results | 33.3 |
| 30 | Ranstam, 2000 [13] | Do you know of any project in your personal proximity in which X occurred during the last 10 years | Fabrication or falsification of data, suppression or selective deletion of data, deceptive design or analysis, deceptive reporting of results, other | 51 |
| 31 |  | Have you been engaged in a project in which X was about to take place during the last 10 years | Fabrication or falsification of data, suppression or selective deletion of data, deceptive design or analysis, deceptive reporting of results, other | 31 |
| 32 | Geggie, 2001 [14] | Do you have first hand knowledge of scientists intentionally X for the purpose of publication | Altering or fabricating data | 10.8 |
| 33 | Meyer,  2004 [15] | Indicate first hand knowledge of X | Falsify data | 15.3 |
| 34 |  |  | Alter data to conform to a certain theory or to increase statistical significance | 27.3 |
| 35 |  |  | Fail to report contrary data and/or results in a manuscript | 69.3 |
| 36 |  |  | Immediately destroy the original database that a study is based upon and, therefore, cannot be used to reconstruct the findings | 6.8 |
| 37 | Gardner,  2005 [16] | Is there X in a study during the past 10 years that you know personally about | Fabricated or falsified data | 7.8 |
| 38 |  |  | Deleted data in an unjustified way | 6.2 |
| 39 |  |  | Deceptive or misleading report of design | 7.2 |
| 40 |  |  | Deceptive or misleading report of data | 7.2 |
| 41 |  |  | Seriously misleading interpretation of results | 6.5 |
| 43 |  |  | At least one of the above | 17.4 |
| 44 | Kattenbraker  2007 [17] | Have you ever witnessed X firsthand | Fabrication of data, e.g., making up results | 8.5 |
| 45 |  |  | Falsification of data, e.g., changing or omitting data points in a study | 13.1 |
| 46 |  |  | Using inadequate or inappropriate research designs, e.g., selecting a research design because of researcher familiarity versus a design appropriate to the study's purpose | 39.2 |
| 47 |  |  | Failing to present data that contradict one's own previous research | 7.8 |
| 48 |  |  | Changing the design, methodology or results of a study in response to pressure from a funding source, e.g., funding source expects certain results so some data are omitted | 7.8 |
| 49 |  |  | Purposely overlooking others' use of flawed data or questionable interpretation of data | 22.2 |
| 50 |  |  | Dropping observations or data points from analyses based on a "gut feeling" that they were inaccurate | 20.3 |
| 51 |  |  | Unnecessarily changing the research design while a study is underway, e.g., switching from qualitative to quantitative methods after data collection has begun | 11.8 |
| 52 |  |  | Choosing a statistical technique for its ability to provide a more favorable outcome | 45.8 |
| 53 |  |  | Reporting only significant findings in published research | 58.8 |
| 54 | Titus,  2008 [18] | In the past three academic years, how many times have you observed or had direct evidence of researchers in your department (or equivalent organizational unit) allegedly committing X | Falsification or Fabrication | 5.2 |

Abbreviations: “Num” = is a progressive number given to each separate question, for reference purposes., “%yes” is the number of respondents who replied affirmatively.

1. Tangney JP (1987) Fraud will out ? Or will it? New Scientist 115: 62-63.

2. Lock S (1988) Misconduct in medical research: does it exist in Britain? British Medical Journal 297: 1531-1535.

3. Simmons RL, Polk HCJ, Williams B, Mavroudis C (1991) Misconduct and fraud in research: social and legislative issues symposium of the Society of University Surgeons. Surgery 110: 1-7.

4. Kalichman MW, Friedman PJ (1992) A pilot study of biomedical trainees' perceptions concerning research ethics. Academic Medicine 67: 769-775.

5. Swazey J, Anderson M, Karen L (1993) Ethical problems in academic research. American Scientist 81: 542-553.

6. Greenberg M, Goldberg L (1994) Ethical challenges to risk scientists: an exploratory analysis of survey data. Science, Technology, and Human Values 19: 223-241.

7. Glick JL (1993) Perceptions concerning research integrity and the practice of data audit in the biotechnology industry. Account Res 3: 187-195.

8. Glick LJ, Shamoo AE (1994) Results of a survey on research practices, completed by attendees at the third conference on research policies and quality assurance. Accountability in Research 3: 275-280.

9.  Eastwood S, Derish P, Leash E, Ordway S (1996) Ethical issues in biomedical research: perceptions and practices of postdoctoral research fellows responding to a survey. Science and Engineering Ethics 2: 89-114.

10. Bebeau MJ, Davis EL (1996) Survey of ethical issues in dental research. Journal of Dental Research 75: 845-855.

11. Rankin M, Esteves MD (1997) Perceptions of scientific misconduct in nursing. Nursing Research 46: 270-276.

12. May C, Campbell S, Doyle H (1998) Research misconduct: A pilot study of British addiction researchers. Addiction Research 6: 371-373.

13. Ranstam J, Buyse M, George SL, Evans S, Geller NL, et al. (2000) Fraud in medical research: an international survey of biostatisticians. Controlled Clinical Trials 21: 415-427.

14. Geggie D (2001) A survey of newly appointed consultants' attitudes towards research fraud. Journal of Medical Ethics 27: 344-346.

15. Meyer MJ, McMahon D (2004) An examination of ethical research conduct by experienced and novice accounting academics. Issues in Accounting Education 19: 413-442.

16. Gardner W, Lidz CW, Hartwig KC (2005) Authors' reports about research integrity problems in clinical trials. Contemporary Clinical Trials 26: 244-251.

17. Kattenbraker MS (2007) Health education research and publication: ethical considerations and the response of health educators. Carbondale: Southern Illinois University. Doctoral thesis.

18. Titus SL, Wells JA, Rhoades LJ (2008) Repairing research integrity. Nature 453: 980-982.
