# Supplementary material for: How Many Scientists Fabricate and Falsify Research? A Systematic Review and Meta-Analysis of Survey Data
Source: PLoS One. 2009 May 29;4(5):e5738. doi: 10.1371/journal.pone.0005738 (PMC2685008; doi:10.1371/journal.pone.0005738)
Supplement: Table S4 — Sensitivity analysis for meta-regression model. (0.07 MB DOC) [file pone.0005738.s004.doc]

Table S4: Sensitivity analysis for meta-regression model.

|  |  | | | | |
| --- | --- | --- | --- | --- | --- |
| **Study Removed** | **Self- / Non-self-** | **Handed / Mailed** | **“Fabricated, Falsified” / “Modified”** | **Medical / other** | **Model R^2^** |
| Kalichman,  1992 [1] | <0.0001 | <0.0001 | 0.002 | 0.0027 | 0.891 |
| Eastwood, 1996 [2] | <0.0001 | <0.0001 | 0.0002 | 0.0001 | 0.919 |
| List,  2001 [3] | <0.0001 | <0.0001 | <0.0001 | 0.0012 | 0.899 |
| Geggie,  2001 [4] | <0.0001 | <0.0001 | 0.0001 | 0.0016 | 0.886 |
| Martinson,  2005 [5] | <0.0001 | 0.0001 | 0.0077 | 0.0194 | 0.833 |
| Henry,  2005 [6] | <0.0001 | <0.0001 | 0.0001 | 0.001 | 0.888 |
| Gardner,  2005 [7] | <0.0001 | <0.0001 | 0.0003 | 0.0041 | 0.877 |
| Tangney,  1987 [8] | <0.0001 | 0.0006 | 0.0004 | 0.0037 | 0.869 |
| Swazey,  1993 [9] | <0.0001 | <0.0001 | 0.0002 | 0.0031 | 0.887 |
| Greenberg,  1994 [10] | <0.0001 | <0.0001 | 0.0003 | 0.0036 | 0.887 |
| Bebeau,  1996 [11] | <0.0001 | <0.0001 | 0.0001 | 0.0207 | 0.889 |
| May,  1998 [12] | <0.0001 | <0.0001 | 0.0001 | 0.0152 | 0.893 |
| Meyer,  2004 [13] | <0.0001 | <0.0001 | 0.0147 | 0.0037 | 0.875 |
| Kattenbraker  2007 [14] | <0.0001 | <0.0001 | 0.0001 | 0.0005 | 0.899 |
| Titus,  2008 [15] | <0.0001 | <0.0001 | 0 | 0.0012 | 0.92 |
| Overall | <0.0001 | <0.0001 | 0.0002 | 0.0022 | 0.872 |

Cells indicate the statistical significance of each independent variable and portion of variance explained by the model when the corresponding study was removed from the sample.

1. Kalichman MW, Friedman PJ (1992) A pilot study of biomedical trainees' perceptions concerning research ethics. Academic Medicine 67: 769-775.

2.  Eastwood S, Derish P, Leash E, Ordway S (1996) Ethical issues in biomedical research: perceptions and practices of postdoctoral research fellows responding to a survey. Science and Engineering Ethics 2: 89-114.

3. List JA, et al. (2001) Academic economists behaving badly? A survey on three areas of unethical behavior. Economic Inquiry 39: 162-170.

4. Geggie D (2001) A survey of newly appointed consultants' attitudes towards research fraud. Journal of Medical Ethics 27: 344-346.

5. Martinson BC, Anderson MS, de Vries R (2005) Scientists behaving badly. Nature 435: 737-738.

6. Henry DA, Kerridge IH, Hill SR, McNeill PM, Doran E, et al. (2005) Medical specialists and pharmaceutical industry-sponsored research: a survey of the Australian experience. Medical Journal of Australia 182: 557-560.

7. Gardner W, Lidz CW, Hartwig KC (2005) Authors' reports about research integrity problems in clinical trials. Contemporary Clinical Trials 26: 244-251.

8. Tangney JP (1987) Fraud will out ? Or will it? New Scientist 115: 62-63.

9. Swazey J, Anderson M, Karen L (1993) Ethical problems in academic research. American Scientist 81: 542-553.

10. Greenberg M, Goldberg L (1994) Ethical challenges to risk scientists: an exploratory analysis of survey data. Science, Technology, and Human Values 19: 223-241.

11. Bebeau MJ, Davis EL (1996) Survey of ethical issues in dental research. Journal of Dental Research 75: 845-855.

12. May C, Campbell S, Doyle H (1998) Research misconduct: a pilot study of British addiction researchers. Addiction Research 6: 371-373.

13. Meyer MJ, McMahon D (2004) An examination of ethical research conduct by experienced and novice accounting academics. Issues in Accounting Education 19: 413-442.

14. Kattenbraker MS (2007) Health education research and publication: ethical considerations and the response of health educators. Carbondale: Southern Illinois University. 178 p.

15. Titus SL, Wells JA, Rhoades LJ (2008) Repairing research integrity. Nature 453: 980-982.
